# Incorporating advanced practice providers into gastroenterology hospitalist teams

**DOI:** 10.1016/j.igie.2023.01.012

**Published:** 2023-02-24

**Authors:** Edward Sun, Sarah Enslin, Janelle Defilippis, Jennifer Ward, Joseph Vicari

**Affiliations:** 1 Donald and Barbara Zucker School of Medicine, Peconic Bay Medical Center, Riverhead, New York, USA; 2 Division of Gastroenterology and Hepatology, Department of Medicine, University of Rochester Medical Center, Rochester, New York, USA; 3 Rockford Gastroenterology Associates, Rockford, Illinois, USA

## Abstract

Gastroenterology (GE) hospitalist teams provide high-quality acute care to hospitalized patients with GI conditions. Advanced practice providers (APPs) are increasingly being incorporated into these teams to improve the quality of care that is delivered. This article details how GE APPs can streamline workflows, increase access to care and timeliness of consultation, and improve interdisciplinary communication. The role of the GE APP is discussed in both the academic practice and private practice settings. Various challenges to adopting the GE APP model are outlined, including the need for formal institution-specific onboarding programs and supportive physician collaborators. Ongoing educational opportunities are key to further developing the medical knowledge and skills of the GE APP. Finally, considerations for designing a rewarding and sustainable GE APP work schedule are discussed.

The increased demand for hospital-based specialty services has led to the development and growth of dedicated gastroenterology (GE) hospitalist teams. These teams provide high-quality acute care to critically ill patients. GE hospitalist teams have demonstrated the ability to improve timeliness of consultation, decrease time to endoscopy for patients who require emergent or urgent procedures, decrease patient length of stay, and improve both patient and provider satisfaction.^1^ The structure of hospitalist teams varies and may contain 1 or more physicians, physician trainees, advanced practice providers (APPs), and other healthcare professionals.

## Benefits of Adding an APP to a GE Hospitalist Team

GE APPs on a GE hospitalist team augment clinical care by increasing the number of patients evaluated and managed on the inpatient service, enabling team members to provide care that maximizes their professional scope of practice, improving continuity of care, providing specialized education to patients and family members, and improving communication among patient care teams (Table 1).^2^ GE APPs can develop acute care skillsets needed to manage GE emergencies through experience, understanding national guidelines and institutional protocols, and specialized training. APPs are also an integral part of periendoscopic patient management, improving patient readiness for procedures and allowing the physicians more time to perform endoscopic procedures.

**Table 1:** Benefits of the advanced practice provider gastroenterology hospitalist

• Improve quality of care
• Timely triage of critical and complex patients
• Improve patient satisfaction
• Provide physicians more time to perform endoscopic procedures
• Improve communication among patient care teams

## Role of the Inpatient GE APP

A team-based approach aims to ensure improved patient care and outcomes.^3^ The role of the APP varies based on the institution, practice setting, number of providers on the GE hospitalist team, and level of experience.

### Academic practice

GE APPs in academic practice play a vital role on the GE hospitalist service. They contribute to the clinical care of patients and augment the educational and research missions of the academic institution.

Clinically, GE APPs can perform both new patient consultations and follow-up visits. They ensure patients are optimized for procedures, and their work helps to minimize delays in procedures by ensuring preprocedure laboratory tests are obtained and addressed appropriately, bowel preparation is ordered and administered in a timely fashion, cardiopulmonary clearance has been obtained when needed, and informed consent has been completed. They serve as liaisons between the GE attending and primary teams and are excellent patient advocates.^4^

In an academic setting, APPs typically work collaboratively with GE fellows, residents, and medical students. In many instances, the GE fellow will triage consults. The GE APP is often assigned consults in a way that optimizes the educational experience of the trainees on the service. The role of the APPs varies depending on their experience, their interest in particular GE subspecialties, specialized skillsets (eg, bedside paracentesis), and the number of team members. This flexibility enables GE services to optimize the role of individual team members.

APPs can also contribute to the academic missions of the GE division. The GE APP may be integral in enrolling patients in clinical trials, device administration, managing databases, analyzing data, writing manuscripts, and presenting at national conferences. GE APPs may also benefit from educational opportunities at their respective institutions that further their own personal and professional development.

### Private practice

In private practice, the GE APP often functions as a leader on the GE hospitalist team, coordinating the workday for the team. Effective execution of daily tasks by the APP improves team efficiency. Similar to academic practice, essential tasks for the GE APP include evaluating new patient consults, rounding on existing patients, and ensuring patients are safe to undergo endoscopic procedures, bowel preparation, and antithrombotic management. GE APPs can assist with transition of care and work closely with outpatient office staff to schedule follow-up appointments, including procedures.^3^

GE APPs should organize their workday to maximize effectiveness and efficiency for the hospitalist team (Table 2). Components of an efficient workday include preround record review and data collection, huddle with collaborating physician to review critical patients and test results, round on existing patients, see new consults, prioritize evaluation of new patients based on severity of illness, communicate throughout the day with the collaborating physician, and round with collaborating physician (Table 3).

**Table 2:** Structure of the gastroenterology advanced practice provider hospitalist workday

• Define workday ○ Daily work hours (8 h vs 10 h) ○ Days per week (4 days vs 5 days) ○ Weekends? ○ Overnight call?
• Patient volume per advanced practice provider
• Number of consults per day
• Team leader

**Table 3:** Advanced practice provider workday plan

• Prerounds record review and data collection
• Morning huddle with collaborating physician
• Round on existing patients
• Evaluate new consults
• Prioritize evaluation of new patients based on severity of illness
• Round with collaborating physician
• Follow-up on procedure, imaging, and laboratory results ordered during the day

## Challenges of Adding APPs to GE Hospitalist Teams

The scope of practice for APPs varies greatly based on state and institutional regulatory boards.^5^ There should be an appropriate leadership and reporting structure in place for the APP to help oversee compliance and licensure. Additionally, the importance of a supportive physician collaborator and mentor cannot be overemphasized and is essential to set up the APP for success.

New-hire APPs often do not have GE training. A formal onboarding program that familiarizes the APP with the practice and institution rules and regulations, provides electronic medical record training, and provides GE-specific education is important. A successful onboarding program will provide APPs with a solid foundation of knowledge.

## Work–Life Satisfaction

Healthcare professionals enjoy working in teams. Individual benefits of healthcare teams are socioemotional benefits, improved job satisfaction, greater role clarity, and enhanced well-being.^6^ Healthcare teams improve cognitive learning experiences for team members.^6^

A key factor in work–life satisfaction is a balanced work schedule. The GE APP work schedule varies among institutions. Some GE APPs work a 10-hour, 4-day work schedule, whereas others work 8 hours, 5 days per week. Differences in work schedules may be based on regional factors and individual preferences of the GE APP. Finding a balance between the needs of the APP and the needs of the practice is key to achieving job satisfaction and success for the APP (Table 4). At the academic institution, because typically GE fellows are on the GE inpatient consult service with the GE APP, there is a certain built-in flexibility for sick days and holidays for the GE APP, which contributes greatly to work–life balance and satisfaction for the APP.

**Table 4:** Work–life satisfaction

• Healthcare teams promote improved job satisfaction, patient outcomes, personal well-being, professional satisfaction, and personal satisfaction
• Advanced practice providers enjoy working in teams
• Improved personal satisfaction improves performance and communication
• Improved personal and professional satisfaction increases organization's ability to retain staff
• Ten-hour, 4-day work weeks and scheduling flexibility are desired by gastroenterology advanced practice provider

## Summary

GE APPs are indispensable members of GE hospitalist teams. They improve efficiency, provide continuity, deliver high-quality care, and improve patient and provider satisfaction. It is important for practice leaders to elucidate the role(s) and tasks for GE APP team members. APPs need a physician mentor who supports their ongoing professional development. Opportunities for growth include the development of a specialized acute care skill set, independently performing procedures such as bedside paracentesis, promotion to leadership positions, participation in hospital committees, and participation in research projects. An ongoing investment in high-quality team-based care, clearly defined roles and responsibilities, appropriate level of oversight, support for ongoing APP professional development, and commitment to work–life satisfaction will ultimately improve APP and physician satisfaction and retention and benefit the overall patient care experience and outcomes.

## DISCLOSURE

*All authors disclosed no financial relationships.*
